# Treatment and Management of Chronic Inflammatory Bowel Diseases: Optimizing Present and Future Therapeutic Choices

**DOI:** 10.3390/jcm11185267

**Published:** 2022-09-06

**Authors:** Lorenzo Bertani

**Affiliations:** 1 Department of Translational Research and New Technologies in Medicine and Surgery, University of Pisa, 56126 Pisa, Italy; lorenzobertani@gmail.com; 2 Department of Surgery, Tuscany North-West ASL, Pontedera Hospital, 56025 Pontedera, Italy

Inflammatory bowel diseases (IBD) are chronic relapsing diseases of the gastrointestinal tract of unknown origin, resulting from an aberrant immune response to microbial and gut-specific antigens in genetically susceptible patients [1]. The two main forms of IBD are Crohn’s disease (CD) [2] and ulcerative colitis (UC) [3], and their prevalence is progressively increasing both in Western world and, particularly, in newly industrialized countries [4].

For several years, drugs such as corticosteroids and aminosalicylates, and immunosuppressive and immunomodulator agents such as azathioprine have been used for the treatment of IBD, with clinical remission as therapeutic goal [5]. Recently, with the advent of more effective therapies such as biologic agents, the herapeutic target has been updated to mucosal healing, with the more ambitious endpoints of histological and biochemical remission [6]. In particular, the therapeutic options currently available for IBD include three classes of biological agents: four anti-TNF (infliximab, adalimumab, golimumab—only for UC- and certolizumab pegol) [7], two anti-integrin (vedolizumab and natalizumab) [8], and two anti-interleukin (IL) agents (ustekinumab and risankizumab) [9]. Moreover, two non-biologic small molecules have recently been approved, with different mechanisms of action: tofacitinib is the first anti-JAK drug approved in IBD (and several more are expected in the next few years), while ozanimod is a sphingosine 1 phosphate modulator [10].

However, the rapidly expanding therapeutic armamentarium opens the issue for the choice of the right drug for the right patient. In fact, about one third of patients starting anti-TNF therapies (which are the most prescribed ones) present a primary non-response in the first 14 weeks of treatment, and up to 50% of the responding patients experience a loss of response over time [11,12,13]. Similar data are available for vedolizumab, where only 40–50% of patients achieve clinical remission, and even less so achieve mucosal healing [14,15,16]. Real-life data of ustekinumab are slightly better in CD [17,18], whereas only one third of patients seem to reach complete clinical remission in UC, despite higher rates of drug persistency in comparison with anti-TNFs [19]. The rate of response seems similar even for tofacitinib [20], whereas, regarding ozanimod, real life studies have not yet been performed, but data of the registration trial highlighted that only a small portion of patients could be considered as a good responder [21].

Another important issue to raise is related to the safety of these therapies. Indeed, although the newer biologics seem to have an extremely low rate of infectious and malignancies complications [22], it is worth noting that the vast majority of patients with IBD are treated with anti-TNFs as the first option, especially for regulatory and reimbursement constraints [23]. Anti-TNF drugs markedly increase the risk of viral, bacterial and fungal infections in patients with IBD, who normally display an incidence rate ranging from 10 to 100 events per 1000 person-years of serious infections [24]. To be able to identify patients responding to treatment early would be crucial for the future to IBD management, in order to choose the best therapeutic option in terms of risk/benefit ratio.

Several biomarkers have been proposed with this purpose. In particular, therapeutic drug monitoring is well accepted in the perspective of a “reactive” management of loss of response [25,26]: high serum levels without anti-drug antibodies could suggest a change mechanism of action, whereas a patient who previously responded to anti-TNF treatment with low serum levels and no antibodies could benefit from a treatment optimization, or could respond to a switch to another anti-TNF in the case of the presence of antibodies [27]. Genetic factors have been studied, such as TREM gene expression [28] or a more complex immune profiling [29], but their application in clinical management would probably be limited to only some tertiary centers. The same considerations could be applied to serum or tissue cytokines [30], although the results of serum oncostatin M in predicting therapeutic non-response to anti-TNFs seem to be particularly interesting [31,32,33,34]. A large multicenter pediatric study suggests the characterization of fecal microbiota in order to predict therapeutic response [35], but even in this case, the clinical implication in everyday clinical practice should be considered limited. One of the most used biomarkers in IBD setting is fecal calprotectin, which is probably the best in indicating endoscopic and histologic activity [36,37]. Several studies highlighted that fecal calprotectin levels after the induction of biological therapies could be used in predicting therapeutic outcome, in anti-TNF [38,39], vedolizumab [39] and ustekinumab [40]. This is very useful in monitoring the treatment, but it is not helpful in choosing one drug over another.

Future therapies are awaited in the next few years [41], and the management of IBD patients will probably further improve. However, the future of research in the IBD setting will surely be focused on the positioning and personalization of the different therapies, increasing effectiveness rates, reducing side effects and optimizing health care resources.
